# HF-OCAQ: Oral comfort assessment in heart failure patients

**DOI:** 10.1371/journal.pone.0319367

**Published:** 2026-07-23

**Authors:** Mei Lu, Runyu Yang, Xiaona Li, Xinkun Wang, Ming Tao

**Affiliations:** 1 Department of Cardiology, Affiliated Hospital of Zunyi Medical University, Zunyi, Guizhou, China; 2 School of Nursing, Zunyi Medical University, Zunyi, Guizhou, China; 3 Department of Hematology, West China Hospital, Sichuan University, Chengdu, Sichuan, China; 4 West China School of Nursing, Sichuan University, Chengdu, Sichuan, China; 5 Department of Nursing, Affiliated Hospital of Zunyi Medical University, Zunyi, Guizhou, China; Alexandria University Faculty of Nursing, EGYPT

## Abstract

Oral discomfort is common among patients with heart failure (HF), with the potential to compromise nutritional intake, communication, and health-related quality of life. Existing oral assessment tools are predominantly clinician-administered and focused on objective pathology, thus failing to adequately capture patients’ subjective experiences of oral comfort. A patient-reported instrument specifically designed for HF populations is lacking. To develop and preliminarily validate a novel PROM, the HF Patients’ Oral Comfort Assessment Questionnaire (HF-OCAQ), designed to multidimensionally assess perceived oral comfort in hospitalized patients with HF. An initial item pool was generated through literature review, qualitative patient interviews, and expert consultation, guided by Kolcaba’s Comfort Theory. Content validity was evaluated using a two-round Delphi process. A pretest was conducted to assess feasibility and clarity. The questionnaire was then administered to hospitalized patients with HF from three general hospitals between June and September 2020. Psychometric comprised assessments of content validity indices, internal consistency reliability, criterion-related validity against the Beck Oral Assessment Scale (BOAS) using Pearson’s correlation, and structural validity via exploratory factor analysis (EFA) using a common-factor model. A total of 190 valid questionnaires were included in the final analysis. The final HF-OCAQ consisted of 24 items. Content validity indices were high (I-CVI range: 0.833–1.000; S-CVI/UA: 0.875; S-CVI/Ave: 0.979). Internal consistency was acceptable (Cronbach’s α = 0.810). Criterion-related validity showed a weak but statistically significant correlation with BOAS scores (r = 0.233, *p* < 0.05), consistent with the conceptual distinction between subjective comfort and objective oral status. Exploratory factor analysis supported a four-factor structure that was theoretically coherent and aligned with the proposed multidimensional construct of oral comfort. The HF-OCAQ was developed and preliminarily validated as a patient-reported instrument for assessing perceived oral comfort in hospitalized patients with HF. The findings provide initial evidence supporting its content validity, internal consistency, and structural validity. Further studies, including confirmatory factor analysis, test–retest reliability, and assessment of responsiveness to clinical change, are warranted before broader clinical and research application.

## Introduction

Heart failure (HF) represents an advanced stage of various cardiovascular diseases and is associated with substantial morbidity and mortality. In recent years, increasing attention has been directed toward oral health as an integral component of comprehensive chronic disease management in this population. A growing body of evidence underscores the importance of integrating oral health care into holistic HF management strategies [1–3]. In addition, The H*ealthy Oral Action Plan* (2019–2025), issued by the National Health Commission explicitly emphasizes the need to strengthen oral health management for patients and to advance the development of clinical oral health care services [4]. Despite these initiatives, the oral health status of hospitalized patients with HF remains suboptimal.

Multiple factors related to disease progression and therapeutic interventions negatively affect oral health in patients with HF. For example, the use of diuretics and strict fluid restriction may reduce salivary secretion, resulting in xerostomia [5]. Decreased saliva impairs the oral cavity’s natural self-cleansing ability, while concurrently, reduced self-care capacity and limited activity tolerance further compromise oral hygiene practices. Consequently, ineffective oral cleaning promotes bacterial proliferation and contributes to an acidic oral microenvironment. Under such conditions, salivary lysozyme activity is diminished, weakening its bactericidal function and facilitating the overgrowth of pathogenic microorganisms, ultimately leading to oral microbiota dysbiosis [6]. These pathological changes adversely affect oral comfort, patients with HF frequently report symptoms such as thick tongue coating, thirst, and taste alterations, which in turn interfere with daily activities, including eating and sleeping.

Scientific and systematic assessment is a prerequisite for effective intervention. However, no validated instrument currently exists to specifically evaluate oral comfort in patients with HF. Existing tools such as the Beck Oral Assessment Scale (BOAS) primarily focus on objective oral health indicators (e.g., mucosal integrity, dentition), whereas visual analogue scales (VAS) are limited to unidimensional symptom intensity assessment. In contrast, oral comfort is a multidimensional and holistic construct encompassing physical sensations (e.g., dryness and pain), psychological responses (e.g., distress related to taste abnormalities), social implications (e.g., communication difficulties), and environmental influences (e.g., treatment-related discomfort). This conceptualization is grounded in Kolcaba’s Comfort Theory [7], which defines comfort as a fundamental outcome of nursing care. Although oral discomfort is not unique to HF, the underlying mechanisms, symptom patterns, and clinical context in patients with HF differ substantially from those observed in the general population or in other chronic diseases. In HF, oral discomfort is frequently influenced by disease-specific factors such as chronic fluid restriction, long-term diuretic therapy, neurohormonal activation, impaired peripheral perfusion, reduced self-care capacity, and repeated hospitalization. These factors may contribute to persistent xerostomia, taste alterations, oral fatigue during speech or drinking, and psychosocial distress associated with symptom burden. Furthermore, oral discomfort in patients with HF is often closely intertwined with overall symptom burden, nutritional intake, sleep quality, medication adherence, and quality of life. Existing oral assessment tools are primarily designed either for objective oral pathology assessment or for general symptom evaluation and therefore may not adequately capture the multidimensional and disease-contextualized oral comfort experiences specific to HF populations. Accordingly, the HF-OCAQ was developed not because oral discomfort is exclusive to HF, but because the clinical manifestations, contributing mechanisms, and patient-perceived impact of oral discomfort in HF may differ meaningfully from those in the general population, thereby requiring a disease-specific patient-reported assessment approach.

Previous studies have occasionally used VAS scores to provide a rough estimation of oral comfort [8]; however, such approaches fail to capture the specific manifestations and overall impact of oral discomfort. Similarly, some oral assessment instruments infer comfort indirectly through evaluation of anatomical abnormalities, rather than directly reflecting patients’ subjective experiences. According to Kolcaba’s Comfort Theory, comfort is a comprehensive state involving four interrelated dimensions physiological, psychological, social, and environmental which provides a robust theoretical framework for assessing subjective experiences. Therefore, incorporating patients’ self-reported perceptions allows for a more accurate understanding of the nature and severity of oral discomfort and facilitates the development of individualized nursing interventions aimed at improving quality of life.

The clinical necessity of addressing oral comfort in HF is underscored by disease-specific mechanisms. Diuretic-induced xerostomia exacerbates physical discomfort through reduced saliva production [5]; metabolic and electrolyte disturbances commonly observed in patients with HF may contribute to taste alterations, oral sensory discomfort, and psychological distress [6]. Furthermore, strict fluid restrictions intensifies thirst perception and challenge patients’ environmental adaptation. These multidimensional discomforts are not adequately captured by existing assessment tools, which remain largely focused on anatomical pathology. The present questionnaire was developed to fill this gap by capturing HF-specific oral comfort experiences across all four theoretical dimensions.

In the present study, oral comfort is defined as a patient-perceived, multidimensional subjective state reflecting the degree of physical ease, sensory acceptability, psychosocial well-being, and functional adaptability related to the oral cavity within a given time frame. Consistent with Kolcaba’s Comfort Theory, oral comfort is conceptualized as a subjective experience rather than an objective clinical condition. It captures patients’ perceptions of oral sensations (e.g., dryness, pain), sensory disturbances (e.g., taste changes), and the extent to which these experiences affect emotional state and daily functioning. Importantly, oral comfort does not aim to measure objective oral pathology or to provide a clinical diagnosis of oral disease. Instead, it reflects how oral conditions regardless of etiology are perceived and experienced by patients in their daily lives. Accordingly, the aim of this study was to develop and validatea patient-reported instrument, the HF-OCAQ, designed to measure the multidimensional nature of oral comfort in patients with HF.

The HF-OCAQ serves as a patient-reported nursing assessment tool for evaluating perceived oral comfort in patients with HF. In its current form, the instrument is designed primarily for: (1) screening for oral discomfort that may warrant further clinical attention; (2) supporting routine nursing assessment by systematically capturing patients’ subjective oral comfort experiences; (3) monitoring patient-reported changes in oral comfort following oral care interventions during hospitalization. The HF-OCAQ is not intended to replace objective oral examinations or to provide a diagnostic assessment of oral disease. Rather, it complements clinical evaluation by focusing on patient-perceived comfort, an aspect often underrepresented in routine care.

## Materials and methods

### Study design and ethical approval

This methodological study was conducted in two sequential phases: (1) questionnaire development and (2) psychometric validation. The study was reviewed and approved by the Biomedical Research Ethics Committee of the Affiliated Hospital of Zunyi Medical University (Approval No. KLLY-2019–040). Written informed consent was obtained from all participants.

### Phase 1: questionnaire development

#### Item pool generation.

Based on Kolcaba’s comfort theory [7] and Locker’s oral health measurement theory [9–12]^,^ combined with interviews conducted with inpatients suffering from HF, a comprehensive framework for assessing oral comfort was developed, encompassing four key dimensions: physical comfort, psychological comfort, social comfort, and environmental comfort. This framework includes 10 primary areas, which are further broken down into 36 specific sub-items. Each item is scored on a 5-point scale, with higher scores indicating greater discomfort. Based on Kolcaba’s Comfort Theory, the HF-OCAQ domains were organized to reflect four interrelated but conceptually distinct dimensions of patient-perceived oral comfort: Physical–sensory comfort: perceived oral sensations such as dryness, pain, soreness, and abnormal oral feelings; Sensory acceptability and function: perceived disturbances in taste, drinking comfort, and speech-related oral function; Perceived oral condition–related distress: patient-reported awareness and distress related to observable oral changes (e.g., tongue coating), reflecting subjective appraisal rather than objective diagnosis; Psychosocial and functional impact: emotional responses (e.g., mood, confidence) and perceived influence of oral discomfort on daily activities and social expression.

These domains are conceptually aligned with Kolcaba’s physiological, psychological, social, and environmental dimensions of comfort, while being specifically adapted to context of oral comfort in patients with HF. Although some items use categorical descriptors (e.g., “type of taste abnormality” or “type of tongue coating color change”), these items are framed as self-reported perceptions rather than clinician-observed signs. Their inclusion is justified by the premise that patients’ awareness and interpretation of such changes contribute directly to their subjective comfort experience and associated distress. Therefore, these items function as indicators of perceived discomfort rather than objective clinical assessments.

#### Literature search and analysis.

A structured literature review was conducted to support the development of the initial item pool. Two master’s-level nursing researchers independently searched the following electronic databases: PubMed, Web of Science, Embase, China National Knowledge Infrastructure (CNKI), and Wanfang Data. The search covered studies published from January 2000 to December 2019.

Search terms included combinations of the following keywords: “heart failure,” “oral comfort,” “oral health,” “xerostomia,” “taste alteration,” “oral symptom,” “patient-reported outcome,” “comfort,” and “nursing assessment.” Both Medical Subject Headings (MeSH) and free-text terms were used where appropriate.

Studies focusing on oral symptoms, oral health-related quality of life, oral care assessment, symptom burden, and patient-reported experiences in patients with HF or other chronic diseases were reviewed. Relevant concepts, symptom domains, and commonly reported oral discomfort manifestations were extracted and categorized according to Kolcaba’s Comfort Theory framework.

After independent screening and evaluation, discrepancies between the two researchers were resolved through discussion within the research team. The findings from the literature review were integrated with qualitative patient interviews to generate the preliminary item pool.

#### Quality control.

The item pool’s build quality control was ensured throughout the process. Two master’s students independently searched, screened, and evaluated the literature, and screened the items. After completing this step, the findings were summarized. In cases of differing opinions, the decisions were made jointly through group discussion. The significance, purpose, and objectives of the study were explained to the experts via phone or email. Questionnaires were issued after obtaining their consent. The design of the expert consultation questionnaire was finalized through group discussion and commissioning. The interface was kept simple and easy to understand, facilitating ease of completion and providing sufficient blank space for experts to exchange opinions.

#### Expert consultation.

Criteria for experts consulted via Letter: (1) Bachelor’s degree or above; (2) Nurse in charge or above; (3) Engaged in basic nursing, oral care or cardiovascular nursing for more than 10 years, knowing relevant knowledge of research; (4) Voluntary participation in this study.

The expert consultation form consisted of two parts. The first part is the expert’s opinion consultation on the questionnaire’s dimension and item setting, and the second part is the basic information of the expert. The first part briefly explains the dimensions of the questionnaire and the theoretical basis for the setting of the items, and asks experts to make judgments on the rationality of the listed items and the relevance of the items to the questionnaire. Rationality was divided into two judgments: “reasonable” and “unreasonable”. If “reasonable” is selected, the relevance of the items is further judged on a 4-point scale (1=”unrelated” to 4 = ”strongly correlated”). The second part collected the basic information of experts, their familiarity with the topic, and the basis for their judgment.

The research group sorted out the relevant materials and sent them to the consulted experts by email. The experts were asked to reply within one week. After an interval of one week, the second round of consultation questionnaires was sent. The expert consultation was terminated when the expert coordination coefficient (Kendall’s W) > 0.5. In the first round, the mean correlation value ≥ 3 points, the coefficient of variation ≤0.4, and the expert judgment “reasonable” were used as the screening indicators. In the second round, more stringent criteria were applied (coefficient of variation ≤ 0.34).

#### Pre-testing and refinement.

A pre-survey was conducted with 20 patients with HF to assess comprehension, relevance, and feasibility. Cognitive debriefing: Patients were interviewed using “think-aloud” techniques and probing questions to understand their interpretation of each item. This process identified ambiguities in terms like “environmental comfort.” Readability: The Flesch-Kincaid Grade Level score was calculated to be 5.2, confirming the questionnaire is easily readable. Item refinement: Based on feedback, items were rephrased for clarity (e.g., “Oral cavity-related articulation comfort evaluation” was simplified to “Speech clarity”). The average completion time was 7 minutes. The clinical version of the Heart Failure Oral Comfort Assessment Questionnaire (HF-OCAQ) was finalized based on this feedback.

The final version of the HF-OCAQ consists of 24 items, each rated on a 5-point Likert scale, with higher scores indicating greater oral discomfort. Response anchors for intensity- or frequency-based items are scored as follows: 1 = not at all/ none; 2 = mild; 3 = moderate; 4 = severe; 5 = extremely severe. For categorical “type” items (e.g., type of taste abnormality or tongue coating change), response options are ordered to reflect increasing perceived discomfort and are coded from 1 to 5 accordingly. Reverse scoring was not applied to any items, as all items were directionally aligned so that higher scores consistently represent greater discomfort. Total score is calculated by summing all item scores, yielding a possible range of 24–120, with higher scores indicating worse oral comfort. Subscale scores are calculated as the sum of items within each domain. Missing data handling: If ≤10% of items are missing, the missing values are replaced by the mean of the completed items within the same domain. Questionnaires with >10% missing items are excluded from scoring and analysis. The predefined threshold for exclusion was set at >10% missing questionnaire items.

### Phase 2: psychometric validation

#### Participants and setting.

Inclusion criteria were as follows: (1) hospitalized patients aged ≥18 years with a diagnosis of HF; (2) New York Heart Association (NYHA) class II–IV; (3) clear consciousness and ability to communicate; and (4) provision of informed consent.

A total of 190 patients with HF in three general hospitals (including 2 tertiary hospitals) from June 2020 to September 2020 were finally enrolled (Table 2). The sample size of the questionnaire reliability and validity test is generally 5–10 times the number of items. With 32 items in the clinical test version, a sample size of 160–320 cases was required.

All participants were diagnosed with HF according to the 2016 European Society of Cardiology (ESC) Guidelines for the diagnosis and treatment of acute and chronic HF. The diagnosis of HF was established by experienced cardiologists based on a comprehensive assessment including: (1) typical symptoms and/or signs of HF; (2) objective evidence of cardiac structural and/or functional abnormalities on echocardiography; and (3) elevated natriuretic peptide levels or a documented history of HF requiring hospitalization. Patients with preserved ejection fraction (HFpEF), mildly reduced ejection fraction (HFmrEF), and reduced ejection fraction (HFrEF) were all eligible for inclusion, provided that they met the above diagnostic criteria and were clinically diagnosed with HF.

Sampling strategy: Participants were recruited using consecutive sampling. All patients admitted to the participating wards during the study period were screened for eligibility and invited to participate if they met the inclusion criteria.

**Fig 1:**
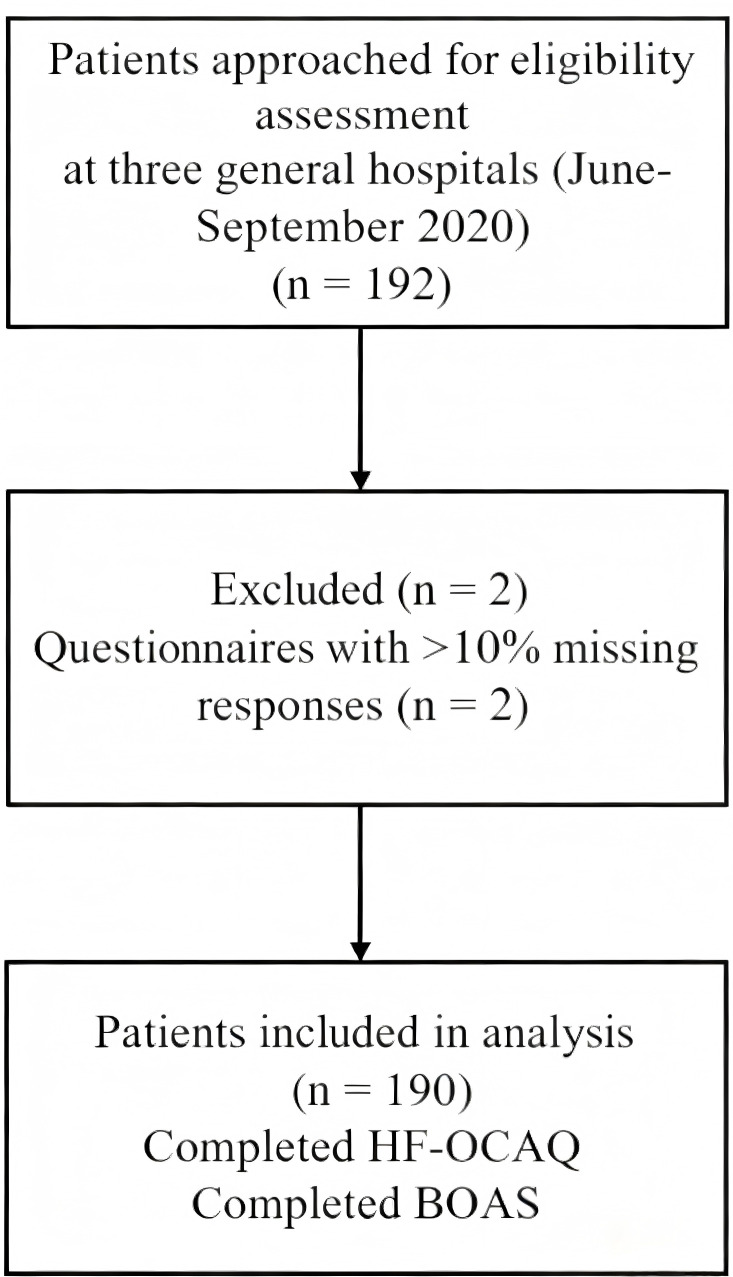
Flow diagram of participant recruitment and inclusion in the validation phase. Between June and September 2020, 192 hospitalized patients with a diagnosis of heart failure were consecutively approached across three general hospitals. Two patients were excluded due to incomplete questionnaire responses exceeding the predefined missing-data threshold (>10% missing items). A total of 190 patients were included in the final psychometric analysis.

During the validation phase, 192 patients hospitalized with a diagnosis of HF were initially approached across three general hospitals between June and September 2020. Of these, 190 patients met the inclusion criteria and provided written informed consent. Two patients were excluded because more than 10% of questionnaire items were missing, according to the predefined missing-data handling criteria (Fig 1).

Additional heart failure-related clinical characteristics were collected from the electronic medical records, including HF phenotype, left ventricular ejection fraction (LVEF), B-type natriuretic peptide (BNP) levels, blood pressure, and major pharmacological treatments. Pharmacological therapies included diuretics, renin–angiotensin–aldosterone system (RAAS) inhibitors, beta-blockers, and electrolyte supplementation. These variables were collected to better characterize the heterogeneity of the HF population and to provide clinical context for interpreting oral comfort experiences. Patients with preserved ejection fraction (HFpEF), mildly reduced ejection fraction (HFmrEF), and reduced ejection fraction (HFrEF) were all eligible for inclusion. HF classification was determined according to the 2016 ESC Guidelines based on LVEF values and clinical diagnosis.

#### Data collection and quality control.

The survey included general data and the HF-OCAQ (see S2 File for full instrument), and the modified BOAS was selected as the standard validity control. A 24-hour recall period was chosen for all items to ensure accurate recall and to capture daily symptom fluctuations commonly experienced during the acute phase of HF management. Before testing, the consent of the ward’s head nurse was obtained. Investigators explained the study purpose and significance to the patients and obtained consent. For patients with vision problems or limited mobility, the researcher assisted in completing the form. The validity and data integrity of the questionnaire were checked on the spot during collection. A total of 192 questionnaires were issued, and 190 valid questionnaires were recovered, with an effective recovery rate of 98.9%. The complete anonymized dataset is provided in S1 Data. To minimize potential confounding effects of severe oral anatomical or pathological conditions, patients with oral tumors or severe oral diseases that could substantially bias subjective oral comfort assessment were excluded. Basic oral hygiene status and anatomical conditions (e.g., dentition status) were evaluated clinically as part of routine nursing assessment. However, the HF-OCAQ was intentionally designed as a patient-reported outcome measure focusing on perceived oral comfort rather than objective oral pathology. Therefore, oral anatomical conditions (such as edentulism) and hygiene status were not directly incorporated into the questionnaire items, but were considered contextual factors when interpreting the results. All participants were required to have clear consciousness and adequate communication ability as part of the inclusion criteria. For patients with visual impairment, advanced age, or limited physical capacity, trained researchers provided standardized assistance by reading the items aloud and recording responses verbatim, without interpretation or guidance. This procedure was applied uniformly to minimize bias related to age, educational background, or functional status.

#### Statistical methods.

The data were verified for accuracy and analyzed using SPSS version 18.0. Sample Size Justification:A post hoc power analysis was performed using G*Power 3.1. With a sample size of 190, an effect size f^2^ = 0.15, and α = 0.05, the achieved power was 90.1% to detect factor loadings above 0.4 in the factor analysis, which is considered sufficient for psychometric studies [13]. Given the primary objective of this study to develop and validate the psychometric properties of the HF-OCAQ analyses focused on reliability and validity testing. Exploratory subgroup analyses examining associations between oral comfort scores and sociodemographic or lifestyle factors (e.g., age, residence, smoking, alcohol consumption) were not prespecified and therefore were not conducted in the present study.

Structural validity was examined using EFA based on a common-factor model, rather than principal component analysis. Given the objective of identifying latent constructs underlying patient-reported oral comfort, principal axis factoring was used for factor extraction. Considering the conceptual relatedness of oral comfort domains, oblique rotation (promax) was applied, allowing correlations among factors. Factor correlations were examined to confirm the appropriateness of oblique rotation. The number of factors to retain was determined using a combination of parallel analysis, scree plot inspection, and theoretical interpretability, rather than reliance on the eigenvalue >1 criterion alone. As HF-OCAQ items are rated on a 5-point Likert scale and therefore ordinal in nature, the factor analysis was conducted using a polychoric correlation matrix, which is more appropriate for ordinal data than Pearson correlations. Sampling adequacy was assessed using the Kaiser-Meyer-Olkin (KMO) measure and Bartlett’s test of sphericity. Factor adequacy and stability were evaluated based on item communalities, factor loadings, and factor overdetermination rather than post hoc statistical power estimates.

#### Psychometric properties assessed.

The following psychometric properties are assessed: Content validity: Measured using the Item-Content Validity Index (I-CVI) and Scale-Content Validity Index (S-CVI/UA, S-CVI/Ave).

Construct validity: Assessed by examining the Pearson correlation coefficient between the HF-OCAQ and the Beck Oral Assessment Scale (BOAS). Pearson’s correlation was used after confirming that both sets of scores met the assumption of normality, as evaluated by the Shapiro-Wilk test (*p* > 0.05). Structural validity:Evaluated via EFA using EFA with varimax rotation (eigenvalue > 1, cumulative variance > 50%, factor loading > 0.4 cutoff) [14]. Internal consistency reliability:Measured using Cronbach’s α coefficient [15].

#### Item analysis.

Critical ratio (CR):The CR decision value [16] was used to screen the items. The upper and lower 27% of the questionnaire score ranking were used as the cut-off value, and the differences between the two groups in each item were compared by independent sample t-test (α = 0.05).

Expert consensus:The concentration degree (mean Mj) and coefficient of variation (Vj) from the Delphi process were used to assess the agreement and dispersion of expert ratings for each item, informing item retention or deletion.

#### Expert consultation metrics.

The initial item pool was developed by the research team based on literature review, theoretical framework construction, and qualitative interviews with hospitalized patients with HF. The research team consisted primarily of nursing researchers and clinicians with experience in cardiovascular nursing, oral care, and patient-reported outcome research. The subsequent expert consultation panel was independent from the initial development team. None of the external experts participated in the original item drafting process. The purpose of the expert consultation stage was to independently evaluate the content relevance, clarity, comprehensiveness, and clinical applicability of the preliminary questionnaire items. During the expert consultation stage, various metrics were examined, including the expert authority coefficient (Cα, a composite score based on knowledge and judgment basis), and Kendall’s W (which measures the degree of consensus among experts).

Construct validity was evaluated using a hypothesis-driven approach, including convergent/divergent validity and known-groups validity analyses. Convergent/divergent validity: We hypothesized that HF-OCAQ scores would show a weak but statistically significant correlation with BOAS, reflecting partial overlap between patient-perceived oral comfort and clinician-assessed oral condition, while remaining conceptually distinct. Known-groups validity: We further hypothesized that HF-OCAQ scores would differ across clinically relevant subgroups expected to vary in oral discomfort severity, including NYHA functional class and oral intake limitations.

## Results

### Phase 1: questionnaire development results

#### Item pool generation and quality control.

Based on Kolcaba’s comfort theory and interviews with patients with HF, an initial comprehensive item pool of 36 items was generated, encompassing four theoretical dimensions: physical, psychological, social, and environmental comfort [9–12,17]. Rigorous quality control procedures, including independent literature screening and resolution of discrepancies through group discussions, ensured the foundational quality and relevance of the initial items.

#### Expert consultation.

A total of 6 experts from 4 hospitals in 3 provinces were selected as members of the expert group (Table 1). According to the results of the first round of expert consultation in Table 2, combined with the screening criteria, “daily leisure activities” were deleted, with two items of “interpersonal relationship with patients”. According to the expert opinion, three items were added: “the degree of influence of the current situation of oral comfort on physical discomfort”, “whether the status of oral comfort causes other physical discomfort”, and “assessment of the current situation of self-confidence brought about by oral comfort”. “Oral language-related language comfort assessment” was divided into two items: “oral cavity-related articulation comfort evaluation” and “oral cavity-related language action comfort evaluation”. Revised “oral speaking-related comfort evaluation” to “degree of language limitations”.

**Table 1:** Basic information on experts.

Project	Classification Number	Number	Percentage (%)
Sex	Male	0	0
	Female	6	100
Age	Over 50 years old	1	16.67
	40-50 years old	4	66.67
	Less than 40 years old	1	16.67
Highest Education	Ph.	0	0
	Master’s Degree	4	66.67
	Bachelor’s Degree	2	33.33
Technical title	Chief Nurse	4	66.67
	Associate Nurse Practitioner	1	16.67
	Nurse Practitioner-in-Charge	1	16.67
Years of working experience	More than 25 years	2	33.33
	15-25 years	2	33.33
	10-15 years	2	33.33

**Table 2:** The advices of experts.

Index	First round of experts’ consultations	Second round of experts’ advice
Personal authority factor	0.75-0.85	0.75-0.9
Group authority coefficient	0.775	0.792
Kendall’s W	0.507	0.616
Cronbach’s α	0.973	0.892
Mj < 3	4 entries	3 entries
Vj > 0.4	3 entries	11 entries

According to the results of the second round of expert consultation in Table 2, combined with the screening criteria, several items were deleted, including “the severity of your taste abnormalities in the past 24 hours”, “the impact of your oral comfort status on the proportion of awakening time in the past 24 hours”, “the impact of your oral comfort status on your overall sleep quality in the past 24 hours”, “the impact of your oral comfort status on your emotional symptoms in the past 24 hours”, “the impact of your oral comfort status on treatment coordination activities in the past 24 hours”, and “The impact of your oral comfort in the past 24 hours on your patient’s relationship with your caregiver”, “The impact of your oral comfort in the past 24 hours on your patient’s relationship with your family”. After two rounds of expert consultation, the clinical version of the “Oral Comfort Assessment Questionnaire for Inpatients with HF” composed of 32 items was finally formed. Following expert consultation, pre-testing, and item analysis, the final validated version of the HF-OCAQ comprised 24 items, rather than the initial 32-item clinical test version. Item analysis was conducted to evaluate the discriminatory ability and contribution of each item to the overall scale. The CR method was used to assess item discrimination. Specifically, participants were ranked according to their total HF-OCAQ scores, and the upper 27% and lower 27% of respondents were defined as the high-score group and low-score group, respectively. Independent-samples t-tests were then performed to compare item scores between the two groups. Items with statistically significant differences (*p* < 0.05) were considered to have acceptable discrimination. In addition, item-total correlations were calculated to examine the relationship between each item and the total scale score. Items with item-total correlation coefficients greater than 0.40 were considered to contribute adequately to the construct measured by the questionnaire. These procedures ensured that each retained item had sufficient discriminatory power and consistency with the overall scale.

#### Pre-testing and cognitive debriefing.

A pre-survey of 20 patients with HF confirmed the questionnaire’s feasibility, with an average completion time of 7 minutes. Cognitive debriefing through “think-aloud” techniques identified ambiguities in terms like “environmental comfort,” leading to item rephrasing for enhanced clarity (e.g., “Oral cavity-related articulation comfort evaluation” was simplified to “Speech clarity”) [18]. The Flesch-Kincaid Grade Level score was calculated to be 5.2, confirming the questionnaire is easily readable [19]. Based on this feedback, the clinical version of HF-OCAQ was finalized for psychometric testing.

### Phase 2: psychometric validation results

#### Participant characteristics.

A total of 190 patients with heart failure in three general hospitals (including 2 tertiary hospitals) from June 2020 to September 2020 were finally enrolled (Table 3). Left ventricular ejection fraction (LVEF) values ranged from 14% to 70% in the study population. The inclusion of patients with LVEF ≥ 50% reflects the presence of HF with preserved ejection fraction (HFpEF) in our cohort. In these patients, the diagnosis of HF was supported by typical symptoms, objective echocardiographic evidence of diastolic dysfunction and/or structural heart disease, and clinical judgment by cardiologists, rather than LVEF alone. Information regarding major comorbidities was collected from the medical records as part of the general clinical characteristics. Common comorbid conditions included hypertension, diabetes mellitus, coronary artery disease, chronic kidney disease, and chronic pulmonary disease. These conditions were recorded because they may potentially influence oral comfort perception, salivary secretion, taste sensation, and oral health status. A high proportion of patients had at least one comorbid condition, reflecting the complex clinical characteristics commonly observed in hospitalized HF populations. Comorbidities such as diabetes mellitus, chronic kidney disease, and chronic pulmonary disease may independently contribute to oral discomfort through mechanisms including altered salivary secretion, oral microbial imbalance, neuropathy, metabolic disturbance, and medication-related adverse effects. Renal function status and the presence of chronic kidney disease were also recorded because renal impairment is common in patients with HF and may potentially influence oral comfort perception and taste sensation.

**Table 3:** Characteristics of the sample (n = 190).

Age in years		
**Mean (*SD*) = 66 ± 12.35 Range 32–87**		
**Gender**	Male	111(58.4%)
	Female	79(41.6%)
**Area**	rural areas	104(54.6%)
	townships	35(18.5%)
	county seat	18(9.3%)
	at or above the city level	33(17.6%)
**Diet Type**	General Diet	186(97.9%)
	Semi-fluid diet	4(2.1%)
	Fluid diet	0
**Smoking status**	Yes	76(40.0%)
	No	114(60.0%)
**Alcohol consumption**	Yes	76(40.0%)
	No	114(60.0%)
**NYHA Functional Class**	Grade 2	16(8.4%)
	Grade 3	146(76.8%)
	Grade 4	28(14.7%)
**Self-care scores**	0-40	7(3.7%)
	41-60	35(18.4%)
	60-99	144(75.8%)
	100	4(2.1%)
**Combined underlying disease**	Yes	167(87.9%)
	No	23(12.1%)
**Sample admission diagnosis**	Range	Mean (SD)
**Beck oral scores**	3-13	7.96 ± 2.27
**Self-care score**	15-100	74.7 ± 16.
**Left ventricular ejection fraction**	14%−70%	39 ± 13.6%
**B-type natriuretic peptide(pg/ml)**	77-35000	6971.69 ± 7650.54
**Systolic blood pressure (mmHg)**	86-181	123 ± 22.82
**Diastolic blood pressure(mmHg)**	45-124	79 ± 14.33

Note: B-type natriuretic peptide (BNP) values were obtained from routine clinical laboratory measurements at admission. Lower BNP levels observed in a small proportion of patients may reflect clinical stabilization after initial treatment, variability related to age, body mass index, or HF phenotype (e.g., HFpEF), and do not preclude a confirmed diagnosis of HF. Among the included patients, the study population represented a heterogeneous HF cohort, including patients with preserved, mildly reduced, and reduced ejection fraction. LVEF values ranged from 14% to 70%, reflecting the broad clinical spectrum of HF phenotypes. Most patients received standard guideline-directed medical therapy for HF, including diuretics, RAAS inhibitors, beta-blockers, and electrolyte supplementation when clinically indicated. Because the primary aim of the present study was psychometric validation of the HF-OCAQ rather than etiological analysis, subgroup analyses according to HF phenotype, cardiac function parameters, or pharmacological treatment were not prespecified. Nevertheless, these clinical variables may influence subjective oral comfort, particularly through mechanisms such as diuretic-induced xerostomia, altered salivary secretion, electrolyte imbalance, and reduced oral intake tolerance.

#### Reliability.

Internal Consistency: The HF-OCAQ demonstrated high internal consistency, with a Cronbach’s α coefficient of 0.810 and a Spearman-Brown coefficient of 0.732 [20].

Inter-Item Correlation: All correlations within the dryness (Q1, Q2) and pain (Q13, Q14, Q15) domains were below the 0.80 threshold for redundancy (highest correlation: r = 0.72 between Q14 and Q15, p < 0.001), supporting the retention of all items as they measure related but distinct constructs (intensity vs. distress) [21].

The HF-OCAQ demonstrated acceptable internal consistency reliability. Cronbach’s alpha for the total scale was 0.81 (95% CI: 0.78–0.84). Subscale reliability estimates were as follows: Factor 1 (Physical-sensory comfort): α = 0.79 (95% CI: 0.75–0.83); Factor 2 (Sensory acceptability and function): α = 0.76 (95% CI: 0.71–0.81); Factor 3 (Perceived oral condition-related distress): α = 0.73 (95% CI: 0.68–0.79); Factor 4 (Psychosocial and functional impact): α = 0.78 (95% CI: 0.74–0.82). Item-total correlations ranged from 0.42 to 0.68 across subscales, indicating that all items contributed meaningfully to their respective constructs. No item deletion resulted in a substantial improvement in subscale reliability.

#### Validity.

Content validity: The content validity analysis demonstrated excellent agreement among experts. The item-level content validity index (I-CVI) ranged from 0.833 to 1.000. The scale-level content validity indices were 0.875 for S-CVI/UA and 0.979 for S-CVI/Ave, all exceeding recommended thresholds and indicating strong content validity [18].

Criterion-related validity: Criterion-related validity was examined by correlating HF-OCAQ scores with BOAS. The Pearson correlation coefficient was 0.233 (*P* < 0.05), indicating a statistically significant but weak association. This magnitude is consistent with theoretical expectations, as the HF-OCAQ assesses patient-perceived oral comfort, whereas BOAS reflects clinician-assessed objective oral status, supporting the conceptual distinctiveness of the two measures.

Structural validity: Structural validity was evaluated using exploratory factor analysis based on a common-factor model. Given the ordinal nature of the 5-point Likert items, analyses were conducted using a polychoric correlation matrix. Sampling adequacy was acceptable (KMO = 0.61), and Bartlett’s test of sphericity was significant (χ^2^ = 1895.78, *p* < 0.001), supporting factorability of the data [22].

The number of factors to retain was determined using parallel analysis, supplemented by scree plot inspection and theoretical interpretability. Parallel analysis supported a four-factor solution, which was consistent with the proposed theoretical framework. Using principal axis factoring with oblique (promax) rotation, four correlated factors were extracted. All retained items demonstrated acceptable communalities (≥ 0.40) and salient factor loadings (≥ 0.40) on their respective factors (Table 4), with minimal cross-loadings. Factor correlations were low to moderate, indicating related but distinct dimensions of oral comfort. The resulting factor structure was theoretically coherent and aligned with Kolcaba’s Comfort Theory [17].

**Table 4:** Factor loading matrix.

Survey Items	Factor1	Factor2	Factor3	Factor4
Q7 A rating of how bothered you have been with mouth sores in the past 24 hours	**0.954**	0.111	−0.083	0.068
Q14 An evaluation of the severity of your mouth pain in the past 24 hours	**0.946**	0.224	−0.041	−0.058
Q13 The duration of your mouth pain in the past 24 hours	**0.934**	0.227	−0.058	−0.074
Q9 An evaluation of your level of distress with abnormal mouth odor in the past 24 hours	**0.867**	−0.025	0.029	0.046
Q1 An evaluation of the severity of your dry mouth in the past 24 hours	**0.843**	0.115	0.243	−0.029
Q15 A rating of how bothered you have been with pain in your mouth in the past 24 hours	**0.839**	0.208	0.021	0.086
Q2 Evaluation of your level of dry mouth in the past 24 hours	**0.775**	0.252	−0.159	−0.021
Q6 An evaluation of the severity of your canker sores in the last 24 hours	**0.753**	−0.071	−0.123	0.229
Q19 Speech motor comfort rating related to your mouth in the last 24 hours	**0.462**	0.154	0.352	−0.18
Q3 Evaluation of the severity of your taste abnormalities in the past 24 hours	0.171	**0.791**	0.269	−0.073
Q5 Evaluation of your sense of taste in the past 24 hours	0.251	**0.788**	−0.056	−0.005
Q17 A drinking comfort rating related to your mouth in the last 24 hours	0.123	**0.773**	0.105	0.012
Q10 The type of color change in your tongue coating in the last 24 hours	−0.03	−0.17	**0.718**	0.203
Q11 An evaluation of the severity of changes in the thickness of your tongue coating in the past 24 hours	−0.186	0.119	**0.685**	0.13
Q26 Evaluation of the impact of your oral comfort on the number of awakenings in the past 24 hours	−0.16	0.153	**0.636**	−0.293
Q12 Evaluation of the degree of distress you have had in the past 24 hours with changes in the coating of your tongue	0.007	0.208	**0.564**	−0.112
Q4 The type of taste abnormality you have in the past 24 hours	−0.102	0.32	**0.551**	−0.201
Q29 An assessment of the degree of distress caused by your oral comfort in the past 24 hours	0.357	0.361	**0.468**	0.111
Q21 The extent to which the current state of oral comfort has affected physical discomfort in the past 24 hours	0.054	0.012	0.027	**0.897**
Q20 Whether your oral comfort in the past 24 hours has caused any other physical discomfort	0.095	−0.014	0.108	**0.894**
Q27 An evaluation of the impact of your oral comfort on your smile expression in the past 24 hours	0.22	0.345	−0.062	**0.831**
Q28 An evaluation of the impact of your oral comfort on your mood in the past 24 hours	0.224	0.165	0.279	**0.557**
Q30 The impact of your oral comfort on activities of daily living in the past 24 hours	0.174	0.487	0.027	**0.503**
Q8 An evaluation of the severity of your abnormal mouth odor in the past 24 hours	−0.1	−0.037	0.351	**0.403**
Eigenvalue	6.27	3.831	2.703	1.502
Cumulative explanatory variation (%)	26.126	42.09	53.352	59.611

Criterion-related validity was assessed using BOAS. A modified version of the BOAS, adapted for routine nursing assessment in hospitalized patients, was used in this study. The modified BOAS retains the original domains of oral assessment (including lips, tongue, mucosa, saliva, and teeth/dentures) while using simplified scoring anchors suitable for bedside nursing evaluation. Higher scores indicate poorer oral condition.

#### Proposed scoring interpretation guidelines.

**Fig 2:**
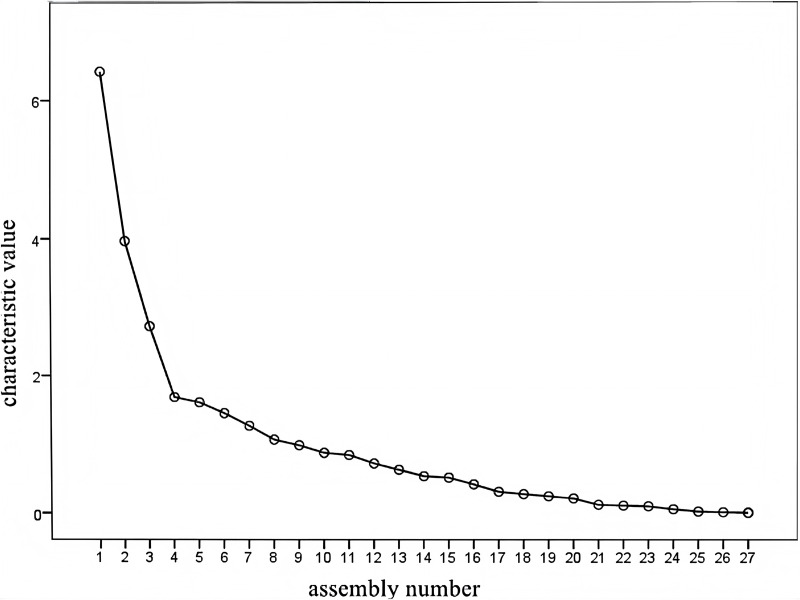
Scree plot of eigenvalues for exploratory factor analysis of the HF-OCAQ (n = 190). The scree plot illustrates the distribution of eigenvalues across extracted factors. A clear inflection point is observed after the fourth factor, supporting the retention of four factors in accordance with parallel analysis and theoretical interpretability.

Based on sample distribution (n = 190), we propose preliminary cut-off scores for the total questionnaire score (range: 24–120): Mild Discomfort: ≤ 56 (≤ 50th percentile); Moderate Discomfort: 57–68 (51st-75th percentile); Severe Discomfort: ≥ 69 (≥ 75th percentile); These cut-offs provide an initial framework for clinical assessment pending future validation with external clinical anchors. Structural validity was evaluated using exploratory factor analysis based on a common-factor model. Given the ordinal nature of the 5-point Likert items, analyses were conducted using a polychoric correlation matrix. Sampling adequacy was acceptable (KMO = 0.61), and Bartlett’s test of sphericity was significant (χ^2^ = 1895.78, *p* < 0.001), supporting factorability of the data. The number of factors to retain was determined using parallel analysis, supplemented by scree plot inspection and theoretical interpretability. As shown in Fig 1, the scree plot demonstrated a clear inflection after the fourth factor, supporting a four-factor solution. Parallel analysis further confirmed the retention of four factors, which was consistent with the proposed theoretical framework (Fig 2).

## Discussion

The development of the HF-OCAQ was guided by Kolcaba’s Comfort Theory, providing a robust multidimensional framework for capturing the complex, subjective experience of oral discomfort in patients with HF [17]. Our findings indicate that the HF-OCAQ is a novel, valid, and reliable instrument that effectively addresses a significant gap in the assessment of patient-centered outcomes in HF care.

### Psychometric properties in context

The strong psychometric properties of the HF-OCAQ support its potential utility in both clinical practice and research.. The internal consistency (Cronbach’s α = 0.810) is comparable to that of well-established patient-reported outcome measures in cardiology and supportive care. For example, the Chinese version of the Memorial Symptom Assessment Scale for Heart Failure (MSAS-HF) reported a similar Cronbach’s α coefficient (0.83) [12]. Furthermore, the four-factor structure identified—Physical Discomfort, Altered Sensation and Function, Objective Oral Signs and Sleep Impact, and Psychosocial and Functional Impact—accounted for 59.611% of the variance. This structure not only aligns coherently with Kolcaba’s theoretical model but also reflects the multifaceted nature of symptom burden seen in other chronic conditions, where factors often encompass physical sensations, functional impacts, and psychosocial consequences [17].

### Discriminant validity and conceptual distinctiveness

The observed weak correlation with BOAS (r = 0.233, *p* < 0.05) is not a limitation but an expected finding that underscores the conceptual distinction between our tool and existing measures. The BOAS is an objective, clinician-reported assessment of oral health pathology, while the HF-OCAQ is a subjective, patient-reported measure of oral comfortand its impact on well-being. This discrepancy between clinician-observed signs and patient-reported symptoms is well-documented in other domains of symptom science [23]. For instance, a patient may have minimal observable oral pathology yet experience severe discomfort from dry mouth or taste changes, profoundly affecting their quality of life. Therefore, the HF-OCAQ provides unique and complementary information, capturing dimensions of the patient experience that are entirely absent from the BOAS and other objective tools.

Respondent burden should be considered when administering patient-reported outcome measures in hospitalized patients with HF, particularly in clinical settings where multiple assessments may be performed concurrently. Several design strategies were used during HF-OCAQ development to improve feasibility and reduce unnecessary burden. The initial 36-item pool was systematically reduced through expert consultation, item analysis, and psychometric evaluation, resulting in a final 24-item version. Pre-testing demonstrated an average completion time of approximately 7 minutes, suggesting acceptable feasibility in hospitalized patients. The HF-OCAQ is intended to function as a targeted, domain-specific assessment tool focusing specifically on oral comfort rather than as a comprehensive quality-of-life instrument. Depending on clinical priorities and patient condition, selective or staggered administration strategies may be considered in practice. Future studies may further optimize the instrument through short-form development using confirmatory factor analysis or item response theory approaches.

### Novelty and clinical utility

The HF-OCAQ fills a critical gap in HF management. While existing instruments like the BOAS or VAS assess singular aspects of oral health, the HF-OCAQ is, to our knowledge, the first tool designed specifically to quantify the multidimensional construct of oral comfort in an HF population. Its development directly addresses the unique oral discomforts precipitated by HF pathophysiology and its management (e.g., diuretic-induced xerostomia, taste alterations).

The clinical utility of the HF-OCAQ is significant. The proposed scoring guidelines offer clinicians a practical framework to systematically identify and triage patients suffering from oral discomfort, facilitating targeted interventions. For example, a patient scoring high on the “Psychosocial and Functional Impact” factor might benefit from counseling and support strategies, while one scoring high on “Physical Discomfort” might require immediate symptomatic relief. By enabling the routine assessment of this neglected aspect of care, the HF-OCAQ empowers healthcare teams to deliver more holistic and personalized patient care, ultimately aiming to improve the quality of life for HF patients.

We acknowledge that the study population was heterogeneous with respect to age, place of residence (rural vs. non-rural), and lifestyle factors such as smoking and alcohol consumption. These factors may influence both oral health status and perceived oral discomfort. However, the primary aim of the present study was instrument development and psychometric validation, rather than etiological analysis of factors associated with oral discomfort. Importantly, the HF-OCAQ was designed to be applicable across diverse patient subgroups by focusing on subjective comfort perception rather than objective oral pathology. The high completion rate (98.9%) and acceptable completion time observed across the study population suggest that demographic and lifestyle differences did not substantially compromise patients’ ability to complete the questionnaire.

The heterogeneous clinical characteristics of patients with HF should also be considered when interpreting oral comfort experiences. HF phenotype, disease severity, neurohormonal activation, and pharmacological treatment may all contribute to variations in perceived oral discomfort. In particular, diuretic therapy and fluid restriction may exacerbate xerostomia through reduced salivary secretion, while electrolyte disturbances and impaired peripheral perfusion may further influence oral sensations and taste perception. In the present study, the primary objective was the development and preliminary psychometric validation of the HF-OCAQ rather than investigation of clinical determinants of oral discomfort. Therefore, associations between HF phenotype, echocardiographic parameters, natriuretic peptide levels, medication use, and oral comfort scores were not formally analyzed. Future studies with larger sample sizes should further explore these relationships to determine whether oral comfort profiles differ across specific HF subgroups and treatment regimens. Comorbid conditions may also play an important role in shaping patients’ oral comfort experiences. For example, diabetes mellitus may contribute to xerostomia, taste disturbance, oral candidiasis, and impaired oral mucosal integrity, while chronic kidney disease may influence oral sensations through metabolic and uremic alterations. Therefore, oral discomfort in hospitalized patients with HF is likely influenced by multiple interacting physiological and treatment-related factors rather than HF alone. Because the present study focused primarily on instrument development and psychometric validation, the independent effects of specific comorbidities on HF-OCAQ scores were not formally analyzed. Future studies with larger and more clinically stratified samples should further evaluate the influence of major comorbidities on oral comfort perception.

Age-related physiological and functional differences may also influence oral comfort perception in patients with HF. Older adults are more likely to experience oral dryness, reduced salivary gland function, impaired dentition, decreased oral self-care ability, and polypharmacy-related oral adverse effects, all of which may contribute to oral discomfort independently of HF itself. In contrast, younger patients may experience different patterns of oral discomfort related more directly to disease burden or treatment-related factors. The broad age range included in the present study reflects the real-world clinical heterogeneity of hospitalized HF populations and was considered important for preliminary psychometric validation of the HF-OCAQ across diverse patient groups. However, age-stratified analyses were not prespecified because the primary objective of this study was instrument development and validation rather than investigation of age-related determinants of oral comfort. Future studies with larger samples should further examine measurement invariance and potential differences in oral comfort profiles across age groups.

The rationale for developing an HF-specific oral comfort instrument is based not on the assumption that oral discomfort occurs exclusively in HF, but rather on the recognition that the mechanisms and clinical context of oral discomfort in HF differ from those in the general population. HF-related fluid restriction, chronic diuretic use, fatigue, reduced physical function, recurrent hospitalization, and complex pharmacological therapy may shape unique patterns of oral discomfort and symptom perception. Moreover, oral discomfort in HF may have distinct clinical implications because it can directly affect fluid intake behavior, nutritional status, treatment adherence, communication, sleep quality, and overall symptom burden. Therefore, a disease-specific patient-reported instrument may provide clinically relevant information that is insufficiently captured by generic oral health or symptom assessment tools.

### Potential clinical applications of the HF-OCAQ

The HF-OCAQ was developed not only as a psychometric instrument, but also as a practical patient-reported assessment tool intended to support symptom-oriented nursing care in hospitalized patients with HF. Oral discomfort is frequently underrecognized in HF management because many symptoms such as xerostomia, taste alteration, oral fatigue, or discomfort during drinking are subjective and may not be readily identified through routine clinical examination alone.

The routine use of the HF-OCAQ may provide several potential clinical benefits. First, it may facilitate early identification of patients experiencing clinically significant oral discomfort who may otherwise not spontaneously report these symptoms. Early recognition may allow healthcare providers to initiate targeted oral care interventions, adjust hydration-related nursing strategies, optimize oral hygiene support, or consider medication-related contributors to oral discomfort.

Second, because oral discomfort may directly influence nutritional intake, fluid intake behavior, medication adherence, communication, sleep quality, and psychosocial well-being, systematic assessment of oral comfort may help clinicians better understand symptom burden and supportive care needs in patients with HF. In particular, severe xerostomia or taste disturbances may contribute to reduced appetite, impaired dietary adherence, or decreased willingness to maintain fluid restriction strategies.

Third, the HF-OCAQ may serve as a longitudinal monitoring tool for evaluating patient-reported responses to oral care interventions during hospitalization. Changes in HF-OCAQ scores over time may help nursing staff assess whether supportive interventions improve subjective oral comfort and overall patient experience.

Importantly, the HF-OCAQ is not intended to replace objective oral examination or specialist dental assessment. Rather, it is designed to complement routine clinical care by systematically capturing the patient’s subjective oral comfort experience, an aspect often underrepresented in conventional HF management.

### Clinical application and future directions

The proposed scoring cut-offs (mild, moderate, severe) for the total questionnaire score, based on sample percentiles, offer a practical starting point for clinical interpretation and triage. However, it is important to note that these are preliminary thresholds. Future research is needed to establish definitive, clinically anchored cut-off values through receiver operating characteristic (ROC) curve analysis against gold-standard criteria, such as expert clinician assessments or patient global impression of change scales.

## Conclusion

The HF-OCAQ developed in this study is grounded in Kolcaba’s Comfort Theory and demonstrates strong reliability and validity. This tool is effective for assessing the oral comfort of hospitalized patients with HF in China, providing a quantitative basis for evaluating their oral comfort. It also serves as an essential resource for nursing staff to gain a deeper understanding of the oral comfort needs of these patients.Future studies should focus on validating this factor structure through confirmatory factor analysis in larger, multi-center cohorts.

Given that this is a self-assessment questionnaire reflecting patients’ subjective experiences, it is recommended to be used alongside objective clinical indicators of oral physiology. This combination allows for a more comprehensive evaluation of oral comfort. By integrating both subjective and objective assessments, healthcare providers can gain a thorough understanding of the oral comfort status of patients with HF, facilitating improved care and more targeted interventions.

In this study, we developed and preliminarily validated HF-OCAQ, a patient-reported instrument grounded in Kolcaba’s Comfort Theory. The findings provide initial evidence supporting the content validity, internal consistency, and structural validity of the HF-OCAQ in a hospitalized HF population. While the results are encouraging, further validation particularly confirmatory factor analysis, test-retest reliability assessment in stable populations, and evaluation of responsiveness to change is required before the instrument can be considered fully established. At present, the HF-OCAQ may be appropriately used as a screening and nursing assessment tool and as an exploratory outcome measure in clinical research focusing on oral comfort in patients with HF. By facilitating systematic identification of patient-perceived oral discomfort, the HF-OCAQ may support individualized oral care strategies and contribute to more holistic symptom management in hospitalized patients with HF.

### Limitations

This study has several limitations. First, the temporal stability (test-retest reliability) of the HF-OCAQ was not assessed. While this is a key psychometric property, we determined it was not methodologically appropriate for our inpatient population due to the dynamic and acute nature of HF symptoms during hospitalization. Patients’ oral comfort status could fluctuate significantly even over 48 hours due to changes in clinical condition and treatments, making it difficult to distinguish measurement error from genuine clinical change [24]. Future validation of the scale’s stability should be conducted in a more clinically stable cohort, such as outpatients.Second, the Delphi process involved a panel of six national experts. While they were highly qualified, expanding the panel to include international experts in future studies could further strengthen the content validity and cross-cultural applicability of the tool. Third, the sample size, while sufficient for the initial factor analysis, was recruited via convenience sampling from hospitals in a specific region of China. This may limit the generalizability of the factor structure. Future multi-center studies involving diverse regions are warranted. Fourth, the factor structure identified through EFA requires validation through CFA in an independent sample. Finally, the use of a 24-hour recall period, while suitable for capturing daily fluctuations in our inpatient setting, may not fully assess longer-term symptom stability in outpatient settings. In addition, although major HF-related clinical characteristics and treatments were recorded, the present study did not perform subgroup analyses according to HF phenotype, cardiac function parameters, or medication categories. These factors may influence oral comfort perception and should be explored in future studies. Furthermore, although information regarding major comorbidities was collected, the present study did not evaluate the independent effects of specific comorbid conditions such as diabetes mellitus or chronic kidney disease on oral comfort scores.

## Supporting information

S1 Data Anonymized dataset for psychometric validation. Complete de-identified dataset used for factor analysis and reliability testing of the Heart Failure Oral Comfort Assessment Questionnaire. Contains 24 questionnaire items, demographic variables (age group, gender, heart function class), and clinical parameters. All direct identifiers removed per PLOS data policy.
(XLS)

S2 File Heart Failure Oral Comfort Assessment Questionnaire (Clinical version). Final 24-item instrument with 5-point Likert scoring system. Chinese/English bilingual version used in the clinical validation study (June–September 2020). This file contains the complete final 24-item HF-OCAQ, including: (1) final item wording (English and Chinese versions); (2) response options and anchors for each item; (3) numeric coding rules for all response options, including categorical “type” items; (4) subscale/domain membership of each item; (5) total and subscale score calculation rules; (6) missing-item handling procedures.
(PDF)

S3 Table Item-to-Domain Mapping and Construct Justification of the HF-OCAQ. (DOCX)

S4 Table Internal consistency reliability and item-total correlations of the HF-OCAQ. (DOCX)

## References

[1] Sanchez P, Everett B, Salamonson Y, Ajwani S, Bhole S, Bishop J, et al. Oral health and cardiovascular care: Perceptions of people with cardiovascular disease. PLoS One. 2017;12(7):e0181189. doi: 10.1371/journal.pone.0181189 28727751 PMC5519046

[2] Rasouli-Ghahroudi AA, Khorsand A, Yaghobee S, Rokn A, Jalali M, Masudi S, et al. Oral health status, knowledge, attitude and practice of patients with heart disease. ARYA Atheroscler. 2016;12(1):1–9. 27114731 PMC4834175

[3] Sanchez P, Everett B, Salamonson Y, Ajwani S, George A. Oral Healthcare and Cardiovascular Disease: A Scoping Review of Current Strategies and Implications for Nurses. J Cardiovasc Nurs. 2017;32(3):E10–20. doi: 10.1097/JCN.0000000000000388 28145980

[4] General Office of the National Health Commission. The National Health Commission issued the action plan for oral health (2019-2025). Beijing: National Health Commission of the People’s Republic of China. 2019.

[5] Lv X, Guang J, Quan HJ. Analysis of influencing factors of thirst in critically ill patients in ICU. Chin J Pract Nurs. 2018;34(9):680–4. doi: 10.3760/cma.j.issn.1672-7088.2018.09.010

[6] Zhou XD, Shi WY. Oral Microecology. 1st ed. Beijing: People’s Health Publishing House. 2013.

[7] Li XM. Introduction to Nursing. 4th ed. Beijing: People’s Health Publishing House. 2017.

[8] Zhu L, Xie S, Li W. Comprehensive nursing intervention on oral comfort in patients with nasopharyngeal carcinoma undergoing radiotherapy. Chin J Mod Nurs. 2014;20(1):44–7. doi: 10.3760/cma.j.issn.1674-2907.2014.01.013

[9] Locker D. Measuring oral health: a conceptual framework. Community Dent Health. 1988;5(1):3–18. 3285972

[10] Quinn C, Dunbar SB, Higgins M. Heart failure symptom assessment and management: can caregivers serve as proxy?. J Cardiovasc Nurs. 2010;25(2):142–8. doi: 10.1097/JCN.0b013e3181bf93a0 20168194 PMC4264792

[11] Albert N, Trochelman K, Li J, Lin S. Signs and symptoms of heart failure: are you asking the right questions? Am J Crit Care. 2010;19(5):443–52. doi: 10.4037/ajcc2009314 19940253

[12] Guo JY, Lü R, Zhang J. Reliability and validity of the Chinese version of the Memorial Symptom Assessment Scale for Heart Failure Patients. Chin J Nurs. 2014;49(12):1448–52. doi: 10.3761/j.issn.0254-1769.2014.12.008

[13] Kline RB. Principles and practice of structural equation modeling. 4th ed. New York: Guilford Press. 2015.

[14] Gao QS. Practical Statistics Methods and Software Operation in Nursing Scientific Research. Shanghai: Shanghai Jiao Tong University Press. 2019.

[15] Zhang YT, Qian XY, Li JJ. Development and reliability and validity test of the Advance Directive Knowledge-Attitude-Practice Questionnaire for the elderly. Chin Nurs Res. 2020;34(23):4154–8. doi: 10.12102/j.issn.1009-6493.2020.23.007

[16] Li Z. Research Methods in Nursing. 2nd ed. Beijing: People’s Health Publishing House. 2018.

[17] Kolcaba K. Comfort theory and practice: A vision for holistic health care and research. New York: Springer Publishing Company. 2003.

[18] Polit DF, Beck CT. The content validity index: are you sure you know what’s being reported? Critique and recommendations. Res Nurs Health. 2006;29(5):489–97. doi: 10.1002/nur.20147 16977646

[19] Babbie ER. The practice of social research. 15th ed. Boston: Cengage Learning. 2020.

[20] Koo TK, Li MY. A Guideline of Selecting and Reporting Intraclass Correlation Coefficients for Reliability Research. J Chiropr Med. 2016;15(2):155–63. doi: 10.1016/j.jcm.2016.02.012 27330520 PMC4913118

[21] Tabachnick BG, Fidell LS. Using multivariate statistics. 7th ed. Boston: Pearson Education; 2019.

[22] Field A. Discovering Statistics Using IBM SPSS Statistics. 4th ed. London: SAGE Publications. 2013.

[23] Cleeland CS. Symptom burden: multiple symptoms and their impact as patient-reported outcomes. J Natl Cancer Inst Monogr. 2007;(37):16–21. doi: 10.1093/jncimonographs/lgm005 17951226

[24] Streiner DL, Norman GR, Cairney J. Health Measurement Scales: A Practical Guide to Their Development and Use. 5th ed. Oxford: Oxford University Press. 2015.

