# Supplementary material for: HF-OCAQ: Oral comfort assessment in heart failure patients
Source: PLoS One. 2026 Jul 23;21(7):e0319367. doi: 10.1371/journal.pone.0319367 (PMC13395405; doi:10.1371/journal.pone.0319367)
Supplement: S2 File — Final 24-item instrument with 5-point Likert scoring system. Chinese/English bilingual version used in the clinical validation study (June–September 2020). This file contains the complete final 24-item HF-OCAQ, including: (1) final item wording (English and Chinese versions); (2) response options and anchors for each item; (3) numeric coding rules for all response options, including categorical “type” items; (4) subscale/domain membership of each item; (5) total and subscale score calculation rules; (6) missing-item handling procedures. (PDF) [file pone.0319367.s002.pdf]

《Questionnaire for the assessment of oral comfort status of hospitalized patients with heart failure》

Note on Theoretical Alignment: The items in this questionnaire were developed to measure the multi-dimensional construct of 'oral comfort' as defined by Kolcaba's Comfort Theory. Items assessing the 'type' of an experience (e.g., taste, coating color) are framed as the patient's own description of their subjective state, serving as indicators of psychological or environmental discomfort.

Questionnaire Number:

question	Score
1.Evaluate the severity of your dry mouth in the past 24 hours<br>None → 1point　Slight→2 points　Somewhat severe → 3 points<br>Severe →4points　Very severe → 5 points	1:
2.Evaluate the level of distress caused by dry mouth in the past 24 hours<br>None → 1 point　Slightly distressing →2 points　Somewhat distressing→3 points<br>Distressing → 4 points　Very distressing → 5 points	2:
3.Evaluate the severity of taste abnormalities in the past 24 hours<br>None → 1 point　Slight → 2 points　Somewhat severe → 3 points<br>Severe → 4 points　Very severe → 5 points	3:
4.Type of taste abnormalities experienced in the past 24 hours<br>None → 1 point　Hypogeusia (reduced sensitivity in taste) → 2 points<br>Dysgeusia (unpleasant taste during eating/drinking) → 3 points<br>Parageusia (abnormal taste perception, e.g., sour tastes bitter) → 4 points<br>Phantogeusia (persistent abnormal taste, e.g., bitter or metallic taste) → 5 points	4:
5.Evaluate the level of distress caused by taste abnormalities in the past 24 hours<br>None → 1 point　Slightly distressing → 2 points<br>Somewhat distressing → 3 points　Distressing → 4 points<br>Very distressing → 5 points	5:
6.Evaluate the severity of mouth ulcers in the past 24 hours<br>Grade 0: Normal mucosa → 1 point<br>Grade 1: Erythema, pain, single ulcer ≤8mm not affecting eating → 2 points<br>Grade 2: More pronounced erythema, increased pain, scattered ulcers, semi-liquid diet possible, ulcer >8mm, ≤15mm → 3 points<br>Grade 3: More severe ulcers and pain, only liquid diet possible, ulcer >15mm→4 points<br>Grade 4: Severe pain, ulcers coalescing, unable to eat, multiple ulcers ≥15mm→5 points	6:
7.Evaluate the level of distress caused by mouth ulcers in the past 24 hours<br>None → 1 point　Slightly distressing →2 points　Somewhat distressing→3 points<br>Distressing → 4 points　Very distressing → 5 points	7:
8.Evaluate the severity of abnormal mouth odor in the past 24 hours<br>None: Barely noticeable bad breath → 1 point<br>Slight: Slight but noticeable bad breath → 2 points<br>Somewhat severe: Moderate bad breath → 3 points<br>Severe: Strong bad breath → 4 points<br>Very severe: Foul odor → 5 points	8:
9.Evaluate the level of distress caused by abnormal mouth odor in the past 24 hours<br>None → 1 point　Slightly distressing → 2 points　Somewhat distressing →3 points<br>Distressing → 4 points　Very distressing → 5 points	9:
10.Type of tongue coating color change in the past 24 hours<br>Thin white → 1 point　Thick white → 2 points　Thin yellow → 3 points<br>Thick yellow → 4 points　Grey-black → 5 points	10:

11.Evaluate the severity of tongue coating thickness change in the past 24 hours<br>None → 1 point Slight → 2 points Somewhat severe → 3 points<br>Severe → 4 points Very severe → 5 points	11：
12.Evaluate the level of distress caused by changes in tongue coating in the past 24 hours<br>None → 1 point Slightly distressing → 2 points Somewhat distressing → 3 points<br>Distressing → 4 points Very distressing → 5 points	12：
13.Duration of oral pain in the past 24 hours<br>None → 1 point A few minutes → 2 points Within a few hours → 3 points<br>Intermittent pain within 24h → 4 points Continuous presence within 24h → 5 points	13：
14.Evaluate the severity of oral pain in the past 24 hours<br>None → 1 point Slight → 2 points Somewhat severe → 3 points<br>Severe → 4 points Very severe → 5 points	14：
15.Evaluate the level of distress caused by oral pain in the past 24 hours<br>None → 1 point Slightly distressing → 2 points Somewhat distressing → 3points<br>Distressing → 4 points Very distressing → 5 points	15：
16.Evaluate your comfort related to drinking in the past 24 hours<br>Very comfortable → 1 point Comfortable → 2 points Average → 3 points<br>Uncomfortable → 4 points Very uncomfortable → 5 points	16：
17.Evaluate your comfort related to speech actions in the past 24 hours<br>Very comfortable → 1 point Comfortable → 2 points Average → 3 points<br>Uncomfortable → 4 points Very uncomfortable → 5 points	17：
18.Did your oral comfort cause other physical discomfort in the past 24 hours<br>None → 1 point Slight → 2 points Somewhat severe → 3 points<br>Severe → 4 points Very severe → 5 points	18：
19.Extent to which oral comfort impacted physical discomfort in the past 24 hours<br>None → 1 point Slight → 2 points Somewhat severe → 3 points<br>Severe → 4 points Very severe → 5 points	19：
20.Evaluate the impact of your oral comfort on awakening frequency in the past 24 hours<br>Positive impact → 1 point Slight positive impact → 2 points No impact → 3points<br>Slight negative impact → 4 points Negative impact → 5 points	20：
21.Evaluate the impact of your oral comfort on your ability to smile in the past 24 hours<br>Positive impact → 1 point Slight positive impact → 2 points<br>No impact → 3 points Slight negative impact → 4 points<br>Negative impact → 5 points	21：
22.Evaluate the impact of your oral comfort on your emotions in the past 24 hours<br>Positive impact → 1 point Slight positive impact → 2 points No impact → 3points<br>Slight negative impact → 4 points Negative impact → 5 points	22：
23.Evaluate the distress caused by negative emotions due to oral comfort in the past 24 hours<br>None →1 point Slightly distressing →2 points Somewhat distressing →3 points<br>Distressing → 4 points Very distressing → 5 points	23：
24.Evaluate the impact of your oral comfort on daily activities in the past 24 hours<br>Positive impact →1 point Slight positive impact →2 points No impact →3points<br>Slight negative impact → 4 points Negative impact → 5 points	24：
