# Supplementary material for: HF-OCAQ: Oral comfort assessment in heart failure patients
Source: PLoS One. 2026 Jul 23;21(7):e0319367. doi: 10.1371/journal.pone.0319367 (PMC13395405; doi:10.1371/journal.pone.0319367)
Supplement: S3 Table — (DOCX) [file pone.0319367.s003.docx]

**Supplementary Table S3. Item-to-Domain Mapping and Construct Justification of the HF-OCAQ**

| Item No. | Item content (abridged) | Assigned domain (revised label) | Kolcaba dimension | Construct role (why it reflects oral comfort) |
| --- | --- | --- | --- | --- |
| Q1 | Severity of dry mouth | Physical-sensory oral comfort | Physiological | Captures patient-perceived intensity of xerostomia, a core bodily sensation directly defining oral comfort. |
| Q2 | Level of dry mouth | Physical-sensory oral comfort | Physiological | Reflects ongoing subjective dryness experience rather than salivary output or objective function. |
| Q6 | Severity of canker sores | Physical-sensory oral comfort | Physiological | Measures perceived mucosal discomfort and irritation, not clinical diagnosis. |
| Q7 | Distress caused by mouth sores | Physical-sensory oral comfort | Physiological | Assesses perceived burden of oral pain on comfort, emphasizing subjective distress. |
| Q13 | Duration of mouth pain | Physical-sensory oral comfort | Physiological | Captures temporal persistence of pain as experienced by the patient. |
| Q14 | Severity of mouth pain | Physical-sensory oral comfort | Physiological | Measures intensity of oral pain as a direct determinant of comfort. |
| Q15 | Degree of being bothered by mouth pain | Physical-sensory oral comfort | Physiological | Emphasizes patient appraisal of pain-related discomfort rather than nociception alone. |
| Q8 | Severity of abnormal mouth odor | Physical-sensory oral comfort | Physiological | Reflects subjective awareness of oral odor and related discomfort, not objective halitosis assessment. |
| Q9 | Distress caused by abnormal mouth odor | Physical-sensory oral comfort | Physiological | Captures emotional and sensory discomfort arising from perceived oral odor. |
| Q3 | Severity of taste abnormality | Sensory acceptability and oral function | Physiological | Measures perceived disruption of taste, a sensory component central to oral comfort. |
| Q5 | Overall sense of taste | Sensory acceptability and oral function | Physiological | Reflects patient-evaluated sensory acceptability of oral intake. |
| Q4 | Type of taste abnormality | Sensory acceptability and oral function | Physiological | Categorical descriptor reflects patient-recognized taste disturbance patterns contributing to discomfort. |
| Q17 | Drinking comfort related to mouth | Sensory acceptability and oral function | Physiological | Assesses functional ease of drinking as perceived by the patient under oral discomfort. |
| Q19 | Speech motor comfort | Sensory acceptability and oral function | Physiological | Captures perceived oral motor comfort affecting speech, not objective articulation testing. |
| Q10 | Type of tongue coating color change | Perceived oral condition–related distress | Psychological | Represents patient awareness and interpretation of visible oral changes, contributing to distress and discomfort. |
| Q11 | Severity of tongue coating thickness change | Perceived oral condition-related distress | Psychological | Measures subjective perception of oral abnormality rather than clinical inspection. |
| Q12 | Distress caused by tongue coating changes | Perceived oral condition-related distress | Psychological | Explicitly captures emotional response to perceived oral changes. |
| Q26 | Impact of oral comfort on awakenings | Perceived oral condition-related distress | Psychological | Reflects how perceived oral discomfort disrupts sleep, indicating sustained discomfort experience. |
| Q29 | Overall distress caused by oral comfort | Perceived oral condition-related distress | Psychological | Global appraisal of oral discomfort burden, integrating multiple perceived symptoms. |
| Q20 | Oral comfort causing other physical discomfort | Psychosocial and functional impact | Social / Environmental | Captures patient-perceived spillover effects of oral discomfort on bodily well-being. |
| Q21 | Oral comfort affecting physical discomfort | Psychosocial and functional impact | Social / Environmental | Measures perceived functional consequences rather than biomedical sequelae. |
| Q27 | Impact on smile expression | Psychosocial and functional impact | Social | Reflects social expressiveness affected by oral discomfort. |
| Q28 | Impact on mood | Psychosocial and functional impact | Psychological / Social | Captures emotional consequences of oral discomfort. |
| Q30 | Impact on activities of daily living | Psychosocial and functional impact | Environmental | Assesses functional adaptation and daily-life interference due to oral discomfort. |

All items in the HF-OCAQ are framed as patient-reported perceptions and appraisals, not clinician-observed oral signs.

Items that may appear to reference observable oral conditions (e.g., tongue coating or taste abnormality types) are included only insofar as patients’ awareness and interpretation of these changes contribute to their subjective comfort or distress, consistent with Kolcaba’s conceptualization of comfort as a holistic, experiential state.
