# Supplementary material for: HF-OCAQ: Oral comfort assessment in heart failure patients
Source: PLoS One. 2026 Jul 23;21(7):e0319367. doi: 10.1371/journal.pone.0319367 (PMC13395405; doi:10.1371/journal.pone.0319367)
Supplement: S4 Table — (DOCX) [file pone.0319367.s004.docx]

Supplementary Table S4. Internal consistency reliability and item–total correlations of the HF-OCAQ

Table S4A：Subscale reliability

| Subscale (Factor) | No. of items | Cronbach’s α | 95% CI for α |
| --- | --- | --- | --- |
| Factor 1: Physical-sensory oral comfort | 9 | 0.79 | 0.75-0.83 |
| Factor 2: Sensory acceptability and function | 4 | 0.76 | 0.71-0.81 |
| Factor 3: Perceived oral condition-related distress | 5 | 0.73 | 0.68-0.79 |
| Factor 4: Psychosocial and functional impact | 6 | 0.78 | 0.74-0.82 |
| Total scale | 24 | 0.81 | 0.78-0.84 |

Table S4B：Item-total correlation

| Item No. | Assigned subscale | Item-total correlation (r) |
| --- | --- | --- |
| Q1 | Physical-sensory oral comfort | 0.54 |
| Q2 | Physical-sensory oral comfort | 0.51 |
| Q6 | Physical-sensory oral comfort | 0.46 |
| Q7 | Physical-sensory oral comfort | 0.62 |
| Q13 | Physical-sensory oral comfort | 0.58 |
| Q14 | Physical-sensory oral comfort | 0.65 |
| Q15 | Physical-sensory oral comfort | 0.61 |
| Q8 | Physical-sensory oral comfort | 0.44 |
| Q9 | Physical-sensory oral comfort | 0.57 |
| Q3 | Sensory acceptability and function | 0.59 |
| Q5 | Sensory acceptability and function | 0.63 |
| Q4 | Sensory acceptability and function | 0.48 |
| Q17 | Sensory acceptability and function | 0.55 |
| Q10 | Perceived oral condition-related distress | 0.47 |
| Q11 | Perceived oral condition-related distress | 0.52 |
| Q12 | Perceived oral condition-related distress | 0.6 |
| Q26 | Perceived oral condition-related distress | 0.45 |
| Q29 | Perceived oral condition-related distress | 0.58 |
| Q20 | Psychosocial and functional impact | 0.64 |
| Q21 | Psychosocial and functional impact | 0.66 |
| Q27 | Psychosocial and functional impact | 0.61 |
| Q28 | Psychosocial and functional impact | 0.49 |
| Q30 | Psychosocial and functional impact | 0.53 |
| Q19 | Psychosocial and functional impact | 0.42 |

Item-total correlations ≥0.40 were considered acceptable, indicating adequate item contribution to their respective subscales.
